# A simple, robust flow cytometry-based whole blood assay for investigating sex differential interferon alpha production by plasmacytoid dendritic cells

**DOI:** 10.1016/j.jim.2022.113263

**Published:** 2022-05

**Authors:** Oliver Sampson, Nicholas Lim, Jemima White, Vinicius Vieira, Henrik Kløverpris, Emily Adland, Chris Conlon, Donal Skelly, Lucy Jones, Lizzie Stafford, Anni Jamsen, Ellie Barnes, Susie Dunachie, John Frater, Paul Klenerman, Marcus Altfeld, Philip Goulder

**Affiliations:** a Peter Medawar Building for Pathogen Research, University of Oxford, South Parks Road, Oxford OX1 3SY, England, UK; b Africa Health Research Institute, Nelson R. Mandela School of Medicine, K-RITH Tower Building, Umbilo Road, Durban, South Africa; c Oxford University Hospitals NHS Foundation Trust, John Radcliffe Hospital, OX3 9DU, England, UK; d Nuffield Department of Clinical Neurosciences, John Radcliffe Hospital, Oxford OX3 9DU, England, UK; e Department of Integrated Sexual Health, Cwm Taf Morgannwg University Health Board, Pontypridd, CF37 1LB, Wales, UK; f Research Department Virus Immunology, Leibniz Institute for Experimental Virology, Hamburg, Germany

**Keywords:** Innate immunity, Immune sex differences, Interferon alpha, Toll-like receptor 7, Plasmacytoid dendritic cell

## Abstract

Central to sex differences observed in outcome from infection and vaccination is the innate immune response, and specifically production of type I interferons by plasmacytoid dendtiric cells (pDCs), the main producers of IFN-α. Evaluation of IFN-α production by pDCs is therefore critical for studies of innate immune function. However, reliable measurement of pDC IFN-α is hampered by reduced cell yields and cytokine production after cryopreservation or after even short delays in stimulating freshly isolated cells. We here describe a simple yet robust method for measuring IFN-α production in pDCs that preserves cell activation and cytokine production through immediate stimulation of whole blood and subsequent maintenance at 37 °C.

## Highlights

• Immediate whole blood stimulation detects differential IFN-α production by pDCs

• Maintaining whole blood samples at 37 °C preserves maximal cytokine production

• 6 h incubation best captures differential pDC activation and cytokine production

• Assay robustness is confirmed by low intra-donor temporal variability

• Cell activation and cytokine production are substantially reduced by cryopreservation

## 1 Introduction

The innate immune response is critical in protecting humans from disease. Through germline-encoded Pattern Recognition Receptors (PRRs), non-specific, broadly acting inflammation is initiated within seconds of microbial exposure that works to limit pathogen spread and prime an adaptive response. The Type-I-interferons (IFN-I), comprising the 13 subtypes of IFN-α, IFN-β and IFN-ω, represent a major component of the innate anti-viral response and are induced upon recognition of viral products by a range of nucleic-acid sensing PRRs; including cytosolic Retinoic acid-Inducible Gene I (RIG-I) and endosomal Toll-Like Receptors (TLRs) (García-Sastre and Biron, 2006). Importantly, TLR7 and TLR8 detect single-stranded RNA, which constitutes the genome of several important viruses, including the pandemic viruses HIV-1 and SARS-CoV-2; both of which are potent inducers of the IFN-α and IFN-β (Doyle et al., 2015; Park and Iwasaki, 2020).

For both these viruses, and many other infections and immunizations, females typically make stronger and more effective immune responses (Klein and Flanagan, 2016; Peckham et al., 2020); an observation that has been linked with greater production of IFN-I by plasmacytoid dendritic cells (pDCs) by females than males in response to TLR7 agonists (Berghöfer et al., 2006; Meier et al., 2009; Seillet et al., 2012; Laffont et al., 2014; Ziegler et al., 2017; Webb et al., 2019; Hagen et al., 2020).

The main producers of IFN-I in both blood and tissues are plasmacytoid Dendritic Cells (pDCs) (Siegal et al., 1999; Fitzgerald-Bocarsly et al., 2008). However, evaluating the role of IFN-I in differential disease outcomes is problematic as IFN-I production by pDCs is substantially reduced by cryopreservation and even short delays in stimulation of freshly isolated peripheral blood mononuclear cells (PBMC) (Meier et al., 2008). Given this sensitivity of pDCs to *ex-vivo* handling, we hypothesised their immediate stimulation, still within the milieu of whole blood as it is being drawn, and subsequent maintenance of the whole blood sample at 37 °C, would maximise pDC function and provide a more accurate representation of true *in-vivo* pDC activity.

## 2 Materials and methods

### 2.1 Ethics statement

For assay development whole blood was taken in sodium heparin vacutainers (BD) from healthy donors (median age 23 yrs; range 19-24 yrs) in accordance with Human Study Protocols approved by the Research Ethics Committee (REC) at Yorkshire & The Humber – Sheffield (GI Biobank Study 16/YH/0247). A second, larger dataset was obtained from participants in accordance with approval from the University of Oxford Medical Sciences Interdivisional Research Ethics Committee (reference R71346/RE001). Written, informed consent was obtained for each participant or, in the case of those under 18 yrs, assent with parental consent.

### 2.2 Stimulation of whole blood

Sodium heparin vacutainers (BD) were prepared in advance to contain 1 ml R10 [RPMI (Gibco) + 10% FBS (Sigma) + 1% Pen/Strep (Sigma) + 1% l-Glutamine (Gibco)] + 2 μg/ml CL097 (Invivogen) + 10 μg/ml Brefeldin A (BFA) (BioLegend) and stored at 4 °C for up to 18 h. To achieve a 1:1 ratio, and 2-fold reagent dilution (final concentrations 1 μg/ml CL097 + 5 μg/ml BFA), 1 ml whole blood was then collected directly into the vacutainer. Except for temperature experiments, vacutainers were then placed in a dry block heater (Fisher) to maintain their temperature at 37 °C. Blood was then transferred to sterile 5 ml FACS tubes and placed in a 37 °C cell culture incubator for requisite incubation durations.

### 2.3 PBMC isolation & cryopreservation

PBMCs were isolated from concurrently collected whole blood by Ficoll (Lymphoprep®) separation in Leukosep tubes (Greiner) (1000 g; 15mins; low deceleration), the mononuclear layer aspirated, and washed twice in 50mls RPMI (Gibco) (700 g; 5mins, then 200 g 7mins) before resuspension in the starting blood volume of R10.

Cells to be cryopreserved were then centrifuged (500 *g*; 3mins) and resuspended in 200 μl per-ml-starting-volume FBS + 10% DMSO to freeze at —80C with a Mr. Frosty® H container (Fisher). Cells remained at —80C for no more than 14 days before being thawed to stimulate.

To thaw, vials were transferred to a 37 °C water bath for 3mins, then cells transferred to Falcon tubes and washed three times in 15 mls R10; the final resuspension being of original blood volume.

### 2.4 Stimulation of PBMC

5 ml FACS tubes were prepared to contain 1 ml R10 + Brefeldin + CL097, as for whole blood (Section 2.2), and 1 ml PBMC added. Tubes were then placed in a tissue culture incubator for requisite incubation.

### 2.5 Cell staining for flow cytometry

Following incubation, whole blood samples were centrifuged (500 *g*; 3mins) and plasma removed for addition of red blood cell lysis solution (BioLegend) for 20 min at room temperature. Tubes were then centrifuged, and resultant white cells washed twice more in PBS + 1% FBS + 20 mM EDTA before transferring to 96-well plates for staining. For PBMC experiments, tubes were centrifuged after incubation, the media aspirated, and cells transferred to 96-well plates for staining. To stain, plates were centrifuged (500 *g*; 2mins) and cells resuspended in 50 μl live-dead + surface stain (antibodies detailed in Supplement Table ST1) for 30mins at 4 °C. Cells were then washed twice in 200 μl PBS + 1% FBS + 20 mM EDTA before incubation in 200 *μ*l Fixation/Permeabilization solution (Fisher) for 30mins 4 °C. Cells were washed twice in Permeabilization buffer (PB; Fisher) and resuspended in 50 *μ*l intracellular stain (antibodies detailed in Supplement Table ST1) for 30mins at 4 °C before two final PB washes and final resuspension in 130 μl PBS. Stained cells were stored at 4 °C for 12-48 h prior to flow cytometry.

### 2.6 Flow cytometry & sample analysis

Samples were run on a BD LSR II Flow Cytometer, and *.fcs* files analysed with FlowJo v10.7.1 (BD). After PBMC, singlet, and live/dead gating, pDCs were defined as: CD56-/CD19-/CD3-/CD11c-/CD14-/CD123+/HLA-DR+ (see gating strategy in Supplementary Fig. S1). The effect of variability in cytometer performance on MFI was controlled for by using the same 6 reference individuals during assay optimisation. Graphs were plotted and analysed in Prism 9.1.2 (Graphpad); two-group continuous variables were analysed with the *t*-test (parametric data) or the Mann-Whitney *U* test (non-parametric data).

### 2.7 Linear model

A linear model to reflect each measured pDC parameter was constructed using R based on log transformed data of interest with sex, age, and timepoint as variables. Variables were then separately regressed out of the model and the goodness of fit to the original model evaluated using the Wald Test to ascertain whether fit was significantly altered by removal of the variable for each parameter. *P*-values corrected for multiple comparisons using the Bonferroni method.

### 2.8 IFN-α ELISA

IFN-α was measured in supernatant collected after stimulation from WB and PBMC samples for measurement with Human IFN Alpha multi-subtype ELISA (PBL Bioscience) as per manufacturers instruction, using a matrix of 50:50 Human AB Serum to R10 for standards dilution. Final concentration was then normalised to pDC number by dividing by cell count.

## 3 Results

### 3.1 Kinetics of the pDC response to CL097 stimulation in whole blood

Since there is an inevitable delay between blood being drawn in the clinic and processed to isolate and stimulate PBMCs in the laboratory, we developed a whole blood assay to stimulate blood as it is being drawn. We first sought to understand the kinetics of the pDC response in immediately stimulated whole blood maintained at 37 °C in 6 healthy donors matched for sex (males *n* = 3, females *n* = 3) and age (median 23 yrs, range 19-24 yrs) and subjected to a range of incubation times from 2 h to 24 h.

**Fig. 1:**
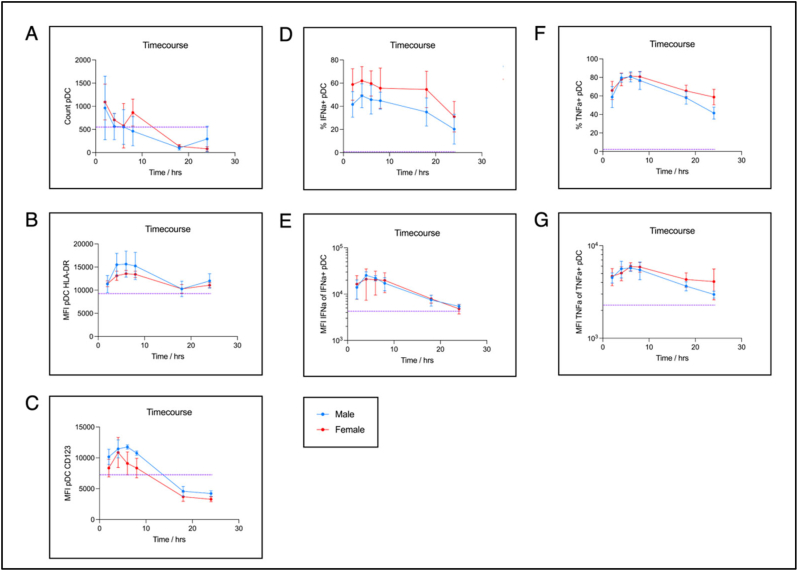
Kinetics of the pDC response to CL097 stimulation in whole blood. Time course of pDC activation and cytokine production in immediately-stimulated whole blood for 6 healthy donors (median age 23 yrs; range 19-24 yrs; male *n* = 3; female *n* = 3). 1 ml blood was drawn into vacutainers containing 1 ml R10 and 2 μg/ml CL097 + 10 μg/ml Brefeldin A (1 μg/ml and 5 μg/ml final conc. respectively) and maintained at 37 °C using a dry block-heater during transport to the laboratory (~30mins) before transfer to loose lid tubes and further incubation in a tissue-culture incubator for indicated times. Red blood cells were then lysed and remaining cells stained as detailed in Methods. Time points were done in duplicate per donor. Dashed purple line represents the average of unstimulated controls processed at 4 h. A) raw count of gated pDCs. B), C), E), G) mean fluorescence intensity (MFI) of gated pDCs of: HLA-DR, CD123, IFN-α, and TNF-α respectively. D), F) percentage of gated pDCs expressing IFN-α and TNF-α respectively. (For interpretation of the references to colour in this figure legend, the reader is referred to the web version of this article.)

As in previous studies there is a marked decrease in pDC numbers with time, with numbers especially low after overnight incubation (Fig. 1A). Activation within pDCs through HLA-DR upregulation showed an increase in MFI to a plateau between 2 h and 8 h, which fell to baseline levels following overnight incubation (Fig. 1B). CD123 upregulation by pDCs had a similar time course (Fig. 1C). The percentage of pDCs expressing IFN-α did not differ significantly after 2-8 h of incubation (Fig. 1D), and remained relatively stable to 18 h, falling to approximately 50% of peak values after 24 h of incubation. The MFI within the IFN-α+ pDC gate showed a similar time course (Fig. 1D). TNF-α production and TNF-α MFI by pDCs both showed similar time courses to IFN-α production (Fig. 1E & F), with peak levels after 4-8 h incubation.

A regression analysis was also performed to identify differences in age, sex, and timepoint on the parameters evaluated in Fig. 1 showed that timepoint significantly impacted all except HLA-DR expression. Whilst sex was also significant for %IFN-α+ pDC and CD123 (Supplementary Table ST2).

In summary, peak pDC activation and cytokine responses were observed 4-8 h post-incubation.

### 3.2 4-8  h incubation best captures sex differential pDC activation and IFN-α production

With several reports in the literature that female pDCs produce more IFN-α than males when stimulated *in-vitro*, and sex being identified as a significant influence in the regression model, we considered these pilot data in Fig. 1, plus Suppl. Fig. S2, to determine whether immune sex differences were apparent even among this small number of study subjects. Whilst the percentage of pDCs producing IFN-α was approximately 50% higher among females, consistent with previous studies (Meier et al., 2009; Webb et al., 2019), the MFI of IFN-α production was not different. HLA-DR expression was markedly higher on male than female pDCs between 4 and 8 h post-stimulation. A similar pattern is seen for CD123, with somewhat increased expression in males, as supported by the regression model (Suppl. Table ST2). By contrast with IFN-α production, TNF-α production by pDCs was broadly similar for the males and females studied at 2-8 h post-stimulation, with increased TNF-α production in females only evident after overnight incubation.

Based on these data, the timepoints after pDC stimulation with the TLR7/8 agonist that best capture sex differential activation of pDCs and IFN-α production are 4-8 h post stimulation. On this basis, and the fact that 6 h post-stimulation represented the peak of TNF-α production by pDCs, we proceeded for the remaining analyses presented using a single timepoint of 6 h post pDC stimulation.

### 3.3 pDC activation and IFN-α production is highest in immediately in-tube stimulated whole blood

Since even a short delay in processing of PBMC has reportedly resulted in significant reductions in pDC function, we next sought to determine whether immediate stimulation of pDCs does best capture both cell activation and cytokine production. Blood was drawn from 4 healthy donors matched for sex (males *n* = 2, females *n* = 2) and age (median 23 yrs, range 19-24 yrs) and subjected to three conditions: i) immediate in-tube stimulation and incubation using dry block-heater for 2 h, followed by transfer to, and further 4 h incubation in, a tissue culture incubator (denoted “WB”), ii) kept at room temperature for 2 h – to simulate transfer from clinic to laboratory – then PBMCs isolated and stimulated for 6 h in a tissue culture incubator (denoted “PBMC”), iii) Remaining PBMCs from ii) were cryopreserved and thawed ≤14 days later for the same 6 h stimulation (denoted “Frozen PBMC”).

**Fig. 2:**
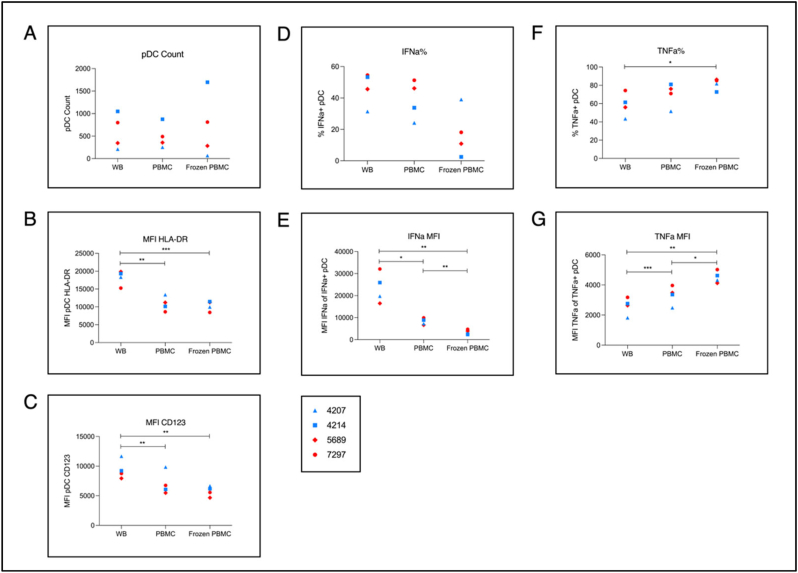
pDC activation and IFN-α production in whole blood compared to PBMC and cryopreserved PBMC. Comparison of immediately in-tube stimulated cells with isolated PBMC and frozen PBMC. Blood was taken from 4 healthy donors (median age 23 yrs; range 19-24 yrs; male *n* = 2; female *n* = 2) and subjected to three conditions: i) immediate stimulation as for Results 2.2; maintained at 37C by dry block heater for 2 h, then incubated for a further 4 h [WB], ii) PBMC isolated after 2 h incubating blood at room temperature then stimulated for 6 h [PBMC], iii) remaining PBMC from ii) cryopreserved and thawed before 6 h stimulation [Frozen PBMC]. Experiments were performed in duplicate per donor. A) raw count of gated pDCs. B), C), E), G) mean fluorescence intensity (MFI) of gated pDCs of: HLA-DR, CD123, IFN-α, and TNF-α respectively. D), F) percentage of gated pDCs expressing IFN-α and TNF-α respectively. Pairwise comparisons performed using Student's paired t-test.

pDC yields in WB were similar to, or somewhat higher than, for PBMC (Fig. 2A). pDC activation through HLA-DR and CD123 expression (Figs. 2B & 2C) was consistently higher in WB than in the other groups. Predictably, the percentage of IFN-α positive pDCs was substantially lower in Frozen PBMC (Fig. 2D), except in one case where pDC counts were extremely low (Fig. 2A). In all cases the IFN-α MFI was lowest in the Frozen PBMC and highest in WB (Fig. 2E). Since the IFN-α MFI was significantly lower for PBMC than WB, despite similar percentages of IFN-α positive pDCs, we sought to investigate whether IFN-α secretion was different. Through ELISA measurement of supernatant collected after stimulation we detected significantly higher IFN-α secretion for WB samples than PBMC (*p* = 0.043; Supplementary Fig. S3). By contrast, TNF-α production followed the converse pattern to IFN-α, with the percentage of TNF-α+ pDCs and TNF-α MFI both being consistently lowest in WB (Figs. 2F & 2G).

### 3.4 Maintaining samples at 37 °C best preserves cytokine production by pDCs

Since use of the dry block heater requires an electricity supply, we next compared the effect on pDCs of maintaining samples at 37 °C during transport, opposed to room temperature, or refrigeration at 4 °C. Blood was drawn into tubes for immediate in-tube stimulation from 4 donors matched for sex (males *n* = 2, females *n* = 2) and age (median 23 yrs, range 19-24 yrs), then incubated for 2 h at either: i) 37 °C, ii) room temperature (20-25 °C) (denoted “RT”), or iii) a transport box maintained at 4 °C (denoted “4C”), before 4 h incubation in a tissue culture incubator to complete 6 h post-stimulation.

**Fig. 3:**
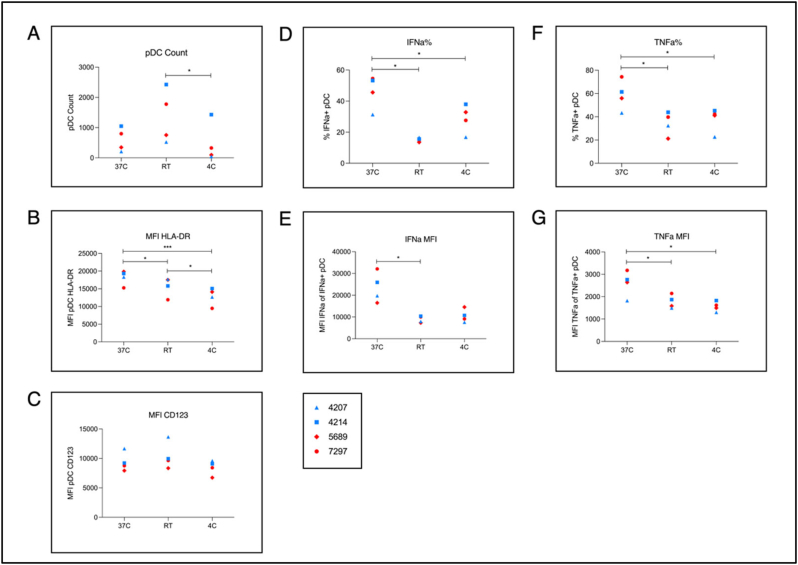
Effect of simulated transport temperature on pDC activation and cytokine response. Comparison of immediately in-tube stimulated blood incubated at either i) 37C, ii) room temperature (20-25C) [RT], or iii) 4C for 2 h before 4 h further incubation in a tissue culture incubator up to 6 h post-stimulation. Conditions were done in duplicate per donor. Donors were matched for age and sex (median 23 yrs; range 19-24 yrs; male *n* = 2; female *n* = 2). A) raw count of gated pDCs. B), C), E), G) mean fluorescence intensity (MFI) of gated pDCs of: HLA-DR, CD123, IFN-α, and TNF-α respectively. D), F) percentage of gated pDCs expressing IFN-α and TNF-α respectively. Pairwise comparisons performed using Student's paired *t*-test.

Unexpectedly, pDC counts were highest in samples incubated at RT in all four donors (Fig. 3A). However, HLA-DR expression, IFN-α production and IFN-α MFI were highest following incubation at 37 °C, as were TNF-α production and TNF-α MFI (Figs. 3D-G).

### 3.5 Low temporal variability between biological repeats confirms longitudinal robustness

**Fig. 4:**
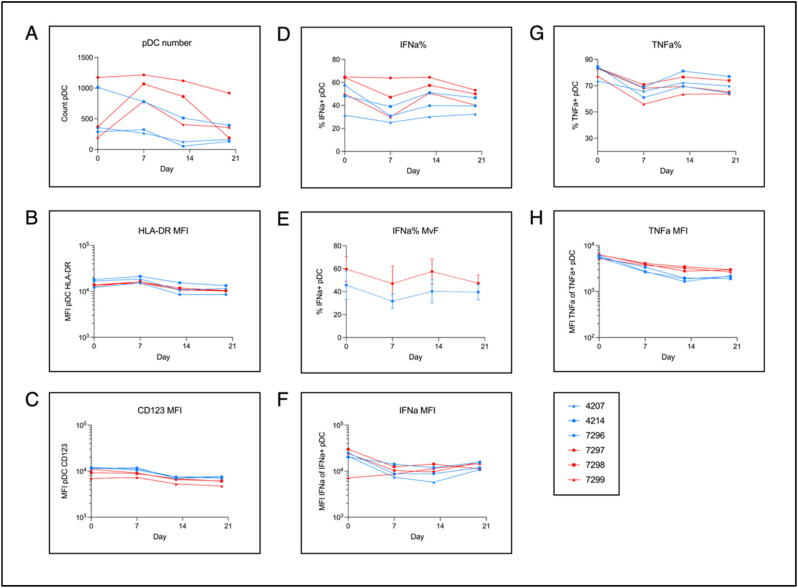
Longitudinal robustness of pDC activation and cytokine production. Assay reproducibility over 4 weeks for 6 healthy donors (median age 23 yrs; range 19-24 yrs; male *n* = 3; female *n* = 3) for immediately in-tube stimulated blood maintained at 37C and incubated for 6  as described in Methods 2.2. Blue symbols indicate male donors and red female donors. A) raw count of gated pDCs. B), C), F), H) mean fluorescence intensity (MFI) of gated pDCs of: HLA-DR, CD123, IFN-α, and TNF-α respectively. D), G) percentage of gated pDCs expressing IFN-α and TNF-α respectively. E) average percentage of pDCs expressing IFN-α for each sex.

When dealing with human populations there is implicit temporal intra-donor heterogeneity. We therefore sought to determine the intra-donor reproducibility of the assay with longitudinal sampling of the same 6 donors as for the time course (Fig. 4).

As expected there was some variability in pDC numbers in the blood of the same individuals sampled at different time points (Fig. 4A). However, the other parameters measured were remarkably consistent for all six donors, illustrated best in the MFI of HLA-DR and CD123 (Fig. 4B and C). In particular, the %IFN-α+ pDCs also shows very limited variability for all donors (Fig. 4D), and remained higher for female donors than male donors across all four weeks (Fig. 4E).

### 3.6 Whole blood assay demonstrates a sex difference in the IFN-α response in a clinical research setting

Since the initial time course and regression successfully identifies sex as a factor in the expression of IFN-α (Suppl. Table ST2), and the small *n* = 6 pilot data supports greater IFN-α production by female pDC's (Fig. 1), we finally sought to confirm the suitability of the whole blood assay in a hospital setting, and that it is sufficiently sensitive to capture the widely reported increased female production of IFN-α in a large cohort.

Blood was taken from 109 individuals (males *n* = 44, females *n* = 65; median 36.5 yrs, range 6-69 yrs) enrolled on a study of health care workers and families, and incubated as described; using the dry heat block to maintain samples at 37C in the clinic after sampling and before transport to the laboratory (median 2 h).

**Fig. 5:**
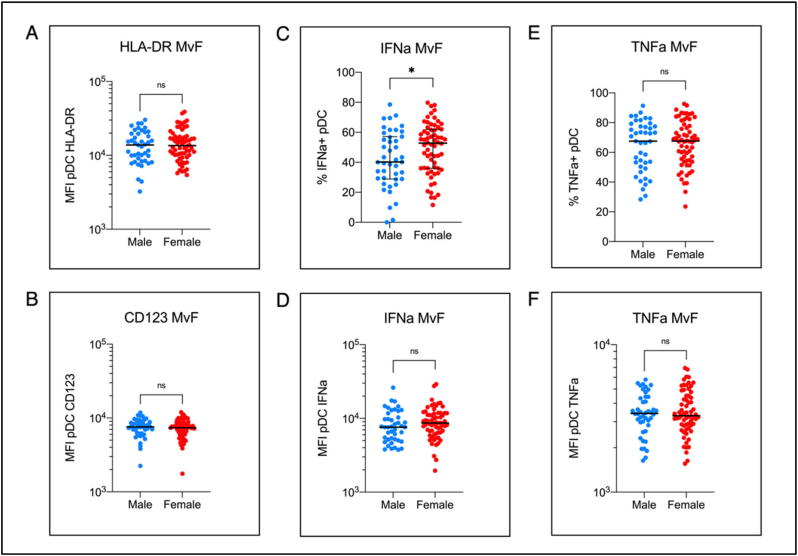
Application of immediate whole blood stimulation in a large clinical research cohort. Application of the whole blood assay in a large dataset (*n* = 109; males *n* = 44; females *n* = 65; age range 6-69 yrs; median 36.5 yrs). A) B), D), F) mean fluorescence intensity (MFI) of gated pDCs of: HLA-DR, CD123, IFN-α, and TNF-α respectively. C), E) percentage of gated pDCs expressing IFN-α and TNF-α respectively. Pairwise comparisons performed using Student's unpaired t-test with Welch's correction for unequal variance.

Indeed, despite a masking of differences in cell activation by the larger numbers (Fig. 5A & B), statistically higher production of IFN-α is still detected in female pDCs (*p* = 0.0394) (Fig. 5C).

Furthermore, in this larger cohort of large age range, comparable pDC IFN-α MFI is still seen for males and females (Fig. 5D) as in the pilot (Fig. 1), as is a comparable percentage of TNF-α + pDCs and TNF-α MFI (Fig. 5E & F).

## 4 Discussion

Since IFN-α production by pDCs plays a central part of the innate immune response it is therefore critical to be able to measure it accurately and reliably. We here developed an assay to address the major setback of working with pDCs; their decreased recovery and subsequent reduction in *in-vitro* activity with increasing time *ex-vivo* (Meier et al., 2008). By drawing blood directly into vacutainers containing media and the TLR7/8 agonist CL097, then maintaining tubes at 37 °C during (simulated) transport to the laboratory, pDC activation was preserved and maximal cytokine production captured. Indeed, the results here show that the delay in transporting blood to the laboratory to then isolate PBMCs incurs a marked reduction in IFN-α production at both the population percentage and single-cell MFI level, which is even more marked when cells were cryopreserved. Mechanistically this decrease appears to be underpinned by a lower relative capacity of pDCs to activate since both HLA-DR and CD123 were reduced concurrently.

Furthermore, we demonstrated the suitability of the assay to a large cohort in a clinical research setting, wherein despite the considerable age range (6-69 yrs), the sensitivity was sufficient to detect the widely-reported increased production of IFN-α by female pDCs achieved through other methods (Berghöfer et al., 2006; Meier et al., 2009; Seillet et al., 2012; Ziegler et al., 2017; Webb et al., 2019; Hagen et al., 2020).

Cells maintained at 37 °C during (simulated) transport displayed better activation through HLA-DR expression, and higher production of IFN-α and TNF-α in both percentage and MFI when compared to those kept at room temperature, or chilled to 4 °C.

Moreover, 37 °C was the only one of the three temperature conditions to detect the increased female %IFN-α+ pDCs previously described in the literature (Berghöfer et al., 2006; Meier et al., 2009; Webb et al., 2019; Hagen et al., 2020). To this end we used this documented increased production if IFN-α by females as a proxy for the ability of the assay to detect differential IFN-α production between groups. Indeed, we were able to detect higher female %IFN-α+ pDCs at each time point during the initial time course. Yet, the same was not seen of IFN-α MFI, suggesting that female pDCs do not produce more IFN-α individually, but that their biallelic expression of TLR7 instead lowers the threshold required for their activation compared to male pDCs. Supporting this is higher expression of HLA-DR and CD123 seen for males at time points where there is the greatest difference between male and female %IFN-α pDCs. However, the sex difference in HLA-DR expression, and to a lesser extent CD123 expression, is mitigated with overnight incubation such that the difference in %IFN-α+ pDCs is reduced. This is again seen when comparing whole blood to PBMC, wherein CD123 MFI was increased in males despite lower male %IFN-α+ pDCs and comparable IFN-α MFI to females.

However, the application of the assay in the larger dataset failed to detect the same differences seen in the time course, which may be attributable to the large range of donor age (6-69 yrs, opposed to 19-24 yrs for assay development) which is known to affect pDC function (Jing et al., 2009).

By using whole blood we draw on the conclusions of Kirchiner et al. (1982) who as early as 1982 note the importance of maintaining the *ex-vivo* milieux of plasma cytokines, as offered by whole blood, for successfully measuring IFN-I, albeit indirectly. Subsequent assays to detect IFN-I directly after specific TLR7 stimulation often include a transfer step from the blood tube before incubation (Coch et al., 2013; Tran et al., 2019), which is impractical for sampling blood away from laboratory sites. Others measure IFN-I through ELISA (Coch et al., 2013; Seeds and Miller, 2011) or SIMOA (Rodriges et al., 2020), which does not allow for the measuring of IFN-I production by any specific cell type. Therefore, a combination method involving in-tube stimulation of whole blood (Rodriges et al., 2020) with immediate incubation at 37 °C (McNerlan et al., 2002) followed by detection of IFN-I *via* FACS (Tran et al., 2019; McNerlan et al., 2002) offers the optimal opportunity to capture maximum cytokine production by pDCs, particularly in paediatric samples where small blood volumes often limit the cell recovery for cryopreservation.

In conclusion, we have here developed an assay that consistently detects differential pDC activation and cytokine production, which is temporally robust despite the fluctuations implicit in work involving human participants. As interest in the role of IFN-I in pathogenic disease outcome grows, we believe this assay to be suited to investigating large cohorts due to its simplicity and reliability, especially for applications in resource-poor settings where time between clinics and laboratory processing are increased.

## Funding statement

This work was supported by the 10.13039/100010269Wellcome Trust [Grant Number: 108869/Z/15/Z (OS); WT104748MA (PG)] and the 10.13039/501100000769University of Oxford Medical Sciences Division COVID19 Fund. Funding bodies had no role in the design, execution, analysis, or submission of the manuscript.

## Declaration of Competing Interest

None to declare.

## Appendix A Supplementary data

Supplementary material Image 1
