# Supplementary material for: A simple, robust flow cytometry-based whole blood assay for investigating sex differential interferon alpha production by plasmacytoid dendritic cells
Source: J Immunol Methods. 2022 May;504:None. doi: 10.1016/j.jim.2022.113263 (PMC9741558; doi:10.1016/j.jim.2022.113263)
Supplement: Supplementary file 1 — Supplementary material [file mmc1.docx]

**Supplement**

***Supplementary Figure S1: Gating Strategy***


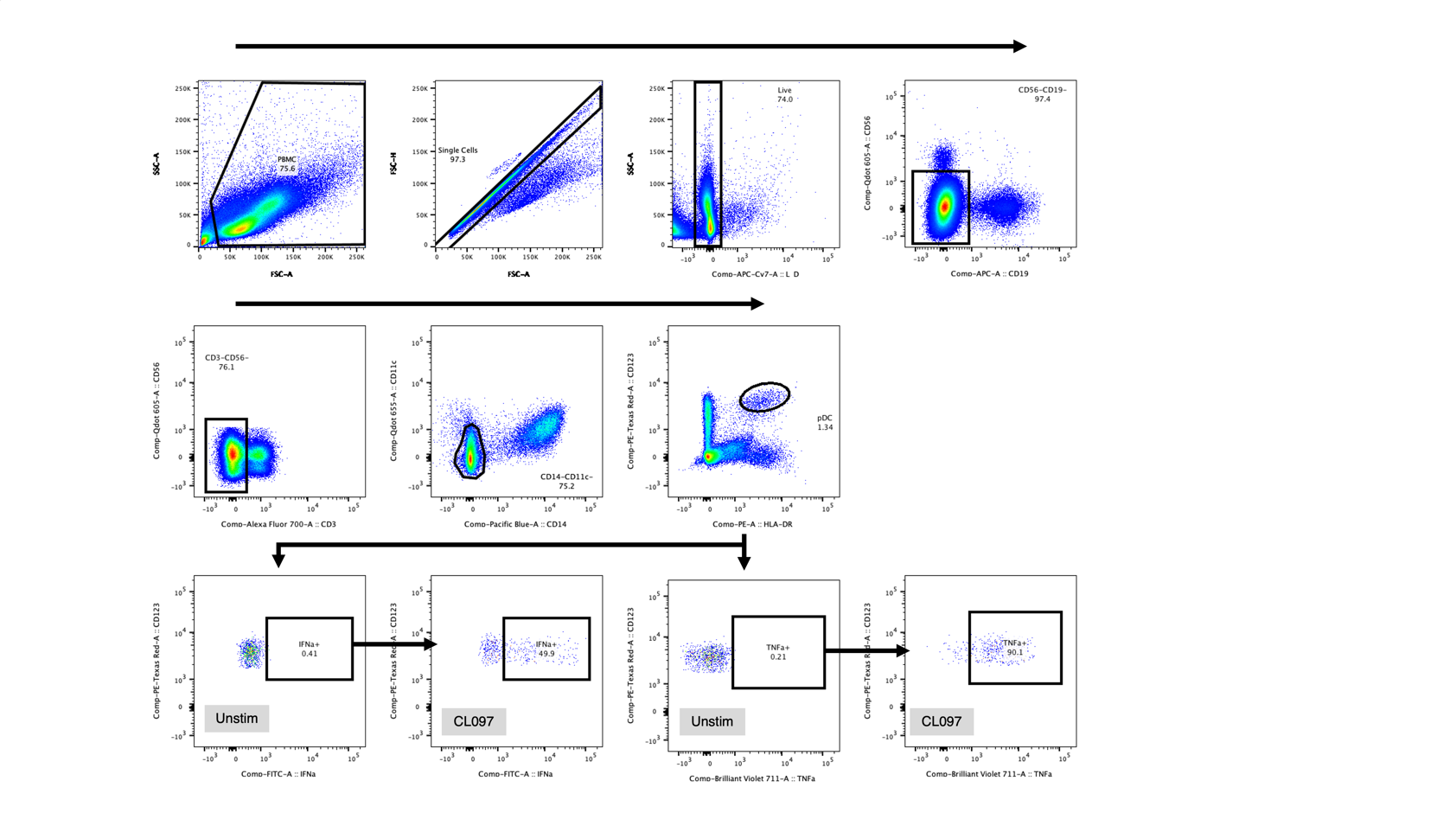
***Supplementary Figure S2: Individual donors from Fig 1.***

*Figure S2 – Individual donor plots for Figure 1 time course. A) Absolute pDC counts; B) MFI of pDC HLA-DR; C) MFI of pDC CD123; D) %IFN-α+ pDCs; E) MFI of IFN-α in IFN-α+ pDCs; F) %TNF-α+ pDCs; G) MFI of TNF-α in TNF-α+ pDCs, Blue symbols indicate a male donor (n=3) and red a female donor (n=3). Dashed purple line represents an unstimulated control processed at 4hrs.*

***Supplementary Figure S3: ELISA measurement of Secreted IFN-α after WB and PBMC stimulation.***

*
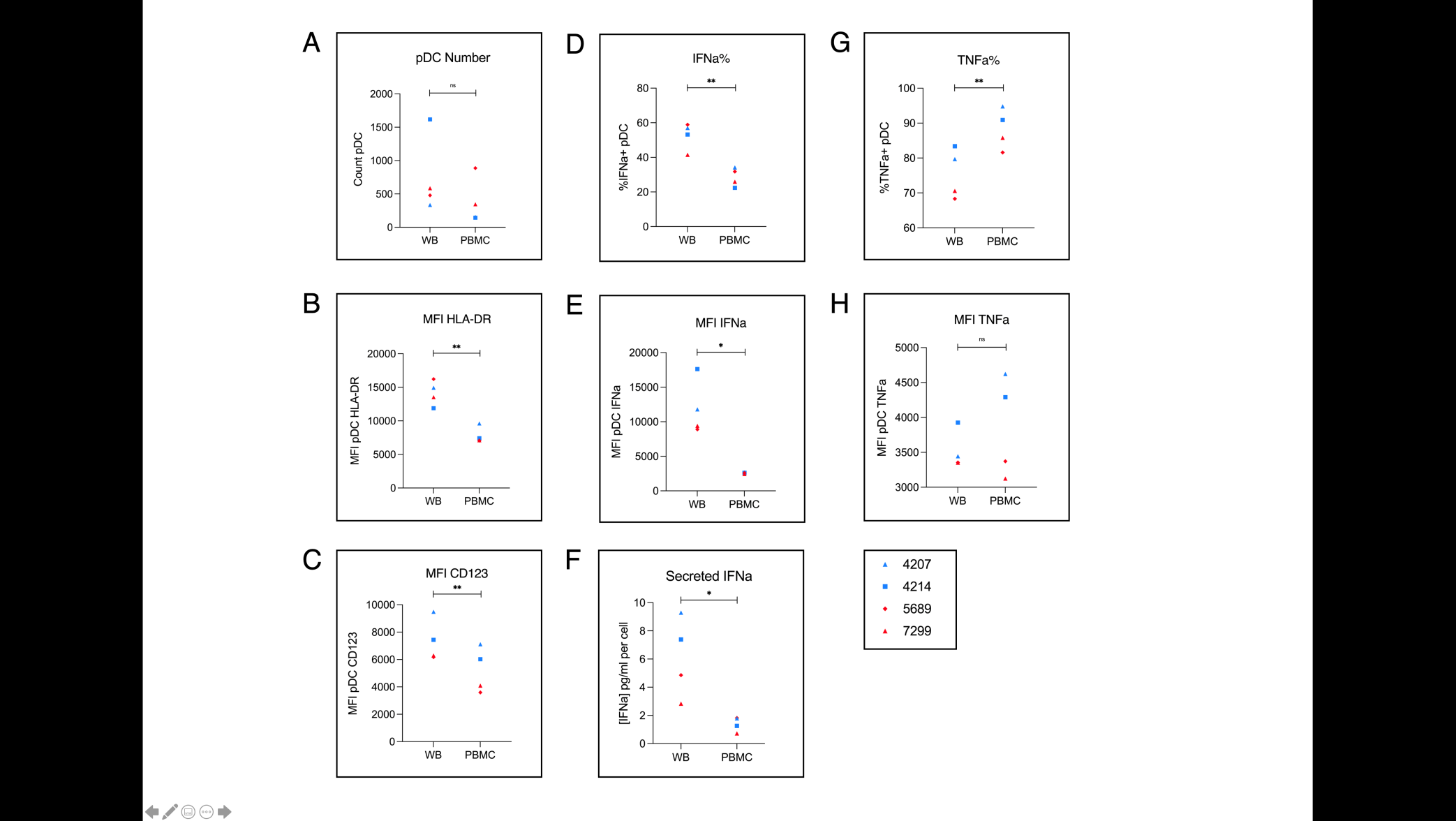
*

*Further comparison of WB and PBMC stimulated pDCs. Blood was taken from 4 healthy donors (median age 24yrs; range 22-25yrs; male n=2; female n=2) and subjected to two conditions: i) immediate stimulation as for Results 2.2; maintained at 37C by dry block heater for 2hrs, then incubated for a further 4hrs [WB], ii) PBMC isolated after 2hrs incubating blood at room temperature then stimulated for 6hrs [PBMC]. Experiments were performed in duplicate per donor. A) raw count of gated pDCs. B), C), E), H) mean fluorescence intensity (MFI) of gated pDCs of: HLA-DR, CD123, IFN-α, and TNF-α respectively. D), G) percentage of gated pDCs expressing IFN-α and TNF-α respectively. F) secreted IFN-α measured by ELISA after concurrent incubation with exclusion of brefeldin A, normalised to cell number. Pairwise comparisons performed using Student’s paired t-test.*

***Supplementary Table ST1: Antibodies used for cell staining***

| **Marker** | **Flurophore** | **Stain** | **Dilution** | **Supplier** | **Catalogue** |
| --- | --- | --- | --- | --- | --- |
| Live/Dead | Near-IR | Surface | 1/1250 | Fisher | L10119 |
| CD56 | BV605 | Surface | 1/50 | BioLegend | 318334 |
| CD19 | APC | Surface | 1/50 | BioLegend | 302212 |
| CD3 | AF700 | Surface | 1/50 | BioLegend | 317340 |
| CD14 | BV421 | Surface | 1/50 | BD | 563743 |
| CD11c | BV650 | Surface | 1/50 | BD | 563404 |
| HLA-DR | PE | Surface | 2/50 | BioLegend | 307606 |
| CD123 | PE-Dazzle594 | Surface | 2/50 | BioLegend | 306034 |
| IFN-α | FITC | Intracellular | 2/50 | Miltenyi | 130-116-872 |
| TNF-α | BV711 | Intracellular | 1/50 | BioLegend | 502940 |

***Supplementary Table ST2: p-values of timepoint linear regression for the three modelled parameters.***

Results of the Linear Model showing adjusted p-values (top; unshaded. Red = significant) and original values (below; grey). P-value represents the significance of the difference in the goodness of fit after regressing out the particular variable – ie. CD123 MFI and %IFN-α+ are significantly altered by regressing out sex, no parameters are altered for age, and all parameters except HLA-DR are altered for time.

| p-value (adjusted) | **Variable** | | |
| --- | --- | --- | --- |
| **Parameter** | Sex | Age | Timepoint |
| pDC Count | 1.00 | 1.00 | 1.71E-14 |
| HLA-DR MFI | 1.00 | 0.17553 | 0.06903 |
| CD123 MFI | 0.003159 | 0.3651 | 6.60E-16 |
| %IFN-α+ | 0.017058 | 0.27894 | 1.65E-07 |
| IFN-α MFI | 1.00 | 1.00 | 9.87E-10 |
| %TNF-α+ | 0.1008 | 1.00 | 4.77E-05 |
| TNF-α MFI | 1.00 | 1.00 | 6.21E-07 |
|  | | | |
| p-value (unadjusted) | Sex | Age | Timepoint |
| pDC Count | 0.757 | 0.6329 | 5.71E-15 |
| HLA-DR MFI | 0.5249 | 0.05851 | 0.02301 |
| CD123 MFI | 0.001053 | 0.1217 | 2.20E-16 |
| %IFN-α+ | 0.005686 | 0.09298 | 5.50E-08 |
| IFN-α MFI | 0.5211 | 0.968 | 3.29E-10 |
| %TNF-α+ | 0.0336 | 0.7152 | 1.59E-05 |
| TNF-α MFI | 0.3532 | 0.8379 | 2.07E-07 |
